# Clinical Relevant Polymorphisms Affecting Clopidogrel Pharmacokinetics and Pharmacodynamics: Insights from the Puerto Rico Newborn Screening Program

**DOI:** 10.3390/ijerph15061115

**Published:** 2018-05-30

**Authors:** Dagmar F. Hernandez-Suarez, Jonnalie C. Tomassini-Fernandini, Angelica Cuevas, Anyelis N. Rosario-Berrios, Héctor J. Nuñez-Medina, Dariana Padilla-Arroyo, Nannette Rivera, Jennifer Liriano, Rocio K. Vega-Roman, Jessicca Y. Renta, Kyle Melin, Jorge Duconge

**Affiliations:** 1 Division of Cardiovascular Medicine, School of Medicine, University of Puerto Rico Medicine Sciences Campus, P.O. Box 365067, San Juan, PR 00936-5067, USA; hector.nunez@upr.edu; 2 Department of Biology, Natural Sciences, University of Puerto Rico Rio Piedras Campus, San Juan, PR 00931, USA; jonnalie.tomassini@upr.edu (J.C.T.-F.); angelica.cuevas30@yahoo.com (A.C.); anyelis.rosario@upr.edu (A.N.R.-B.); nannette.rivera1@upr.edu (N.R.); jenniferliriano14@gmail.com (J.L.); 3 Pharmaceutical Sciences Department, University of Puerto Rico Medical Sciences Campus, San Juan, PR 00936, USA; dariana.padilla@upr.edu (D.P.-A.); jorge.duconge@upr.edu (J.D.); 4 Department of Biology, Natural Sciences, University of Puerto Rico Bayamon Campus, Bayamon, PR 00959, USA; rocio.vega@upr.edu; 5 Department of Biochemistry, University of Puerto Rico Medical Sciences Campus, San Juan, PR 00936, USA; jessicca.renta@upr.edu; 6 Department of Pharmacy Practice, University of Puerto Rico Medical Sciences Campus, San Juan, PR 00936, USA; kyle.melin@upr.edu

**Keywords:** clopidogrel, pharmacogenetics, Puerto Ricans, genotypes, allele frequency

## Abstract

*Background:* Variations in several clopidogrel-pharmacogenes have been linked to clopidogrel response variability and clinical outcomes. We aimed to determine the frequency distribution of major polymorphisms on *CYP2C19*, *PON1*, *ABCB1* and *P2RY12* pharmacogenes in Puerto Ricans. *Methods*: This was a cross-sectional, population-based study of 200 unrelated “Guthrie” cards specimens from newborns registered in the Puerto Rican newborn screening program (PRNSP) between 2004 and 2014. Taqman^®^ SNP assay techniques were used for genotyping. *Results:* Minor allele frequencies (MAF) were 46% for *PON1* (rs662), 41% for *ABCB1* (rs1045642), 14% for CYP2C19*17, 13% for CYP2C19*2, 12% for *P2RY12*-H2 and 0.3% for CYP2C19*4. No carriers of the CYP2C19*3 variants were detected. All alleles and genotype proportions were found to be in Hardy–Weinberg equilibrium (HWE). Overall, there were no significant differences between MAFs of these variants in Puerto Ricans and the general population (*n* = 453) of the 1000 Genome project, except when comparisons to each individual parental group were performed (i.e., Africans, Europeans and East-Asians; *p* < 0.05). As expected, the prevalence of these markers in Puerto Ricans most resembled those in the 181 subjects from reference populations of the Americas. *Conclusions:* These prevalence data provide a necessary groundwork for future clinical studies of clopidogrel pharmacogenetics in Caribbean Hispanics.

## 1. Introduction

Clopidogrel, generic for Plavix^®^, is commonly used to prevent atherothrombotic events in patients with cardiovascular disease. Despite the advent of newer P2Y_12_ adenosine diphosphate (ADP) receptor blockers (e.g., prasugrel, ticagrelor, cangrelor), clopidogrel remains the mainstay of antiplatelet therapy in the Commonwealth of Puerto Rico [1]. The low cost and easy availability makes clopidogrel the best choice for secondary prevention of heart attack and strokes in cardiovascular patients who are at high risk for thrombotic events and ischemic complications, including those with acute coronary syndrome (ACS), stroke, peripheral artery disease (PAD), carotid stenting and cerebral endovascular interventions [1,2,3,4,5]. 

As a pro-drug, the active thiol metabolite is responsible for the effective inhibition of ADP-induced platelet activation and aggregation by selectively and irreversibly blocking the purinergic P2Y_12_ receptor on platelets, a two-step bioactivation process that is mainly mediated by the hepatic drug-metabolizing enzyme *CYP2C19* [6]. Although significant benefits of dual antiplatelet therapy (DAPT) with aspirin have been demonstrated in large clinical trials [7,8], the occurrence of major adverse cardiovascular events (MACE) remains a serious clinical problem [9]. To date, several studies have reported the negative impact of clopidogrel response variability on clinical outcomes, mostly for poor responsive patients [10,11]. Indeed, up to 50% of all treated patients might be “resistant” to clopidogrel, a term that is frequently used to designate those individuals with high on-treatment platelet reactivity (HTPR) [12]. Multiple factors have been suggested to contribute to this lack of response [13]. However, the unique genetic background of each individual has lately emerged as a potential determinant of clopidogrel responsiveness [14]. 

On the other hand, many studies have also highlighted the clinical utility of genotyping patients on clopidogrel [15]. Accordingly, in 2010, the U.S. Food and Drug Administration (FDA) issued a black box warning on Plavix^®^ to inform both patients and healthcare providers about the expectation that *CYP2C19* poor metabolizers are at higher risk of treatment failure [16]. Other relevant pharmacogenes (e.g., *P2RY12, PON1* and *ABCB1*) have also been postulated in previous reports as potential predictors of clopidogrel response [17]. 

The complex genomic structure and admixture pattern of Puerto Ricans gives rise to a richer repertoire of combinatorial genotypes. This unique admixture could have a critical impact on important pharmacological, biochemical and physiological pathways, including those concerning the clopidogrel pharmacokinetics and pharmacodynamics [18]. In previous studies by our group, we have partially described the frequency of polymorphisms affecting clopidogrel pharmacokinetics, but they are limited to certain common variants in *CYP2C19* and *PON1*. Accordingly, a more comprehensive assessment of clopidogrel-associated pharmacogenes in Puerto Ricans is lacking. Thus, we aimed to identify the frequency distribution (prevalence) of major genetic polymorphisms on *CYP2C19*, *PON1*, *ABCB1* and *P2RY12* pharmacogenes in Puerto Ricans by using a population-based, stratified-by-region, representative sample of specimens from the local newborn screening program (PRNSP). 

## 2. Materials and Methods

### 2.1. Study Design and Ethics

This was a cross-sectional study of prevalence of “Guthrie” cards specimens from newborns registered in the Puerto Rican newborn screening program (PRNSP) between 2004 and 2014. A category 4 exemption was granted by the local institutional review board (Protocol A4070316) of the University of Puerto Rico Medical Sciences Campus (UPR MSC) to conduct the study. The study was also authorized by the advisory board of the PRNSP. 

### 2.2. Sample and Data Collection

Two hundred (*n* = 200) unrelated genomic DNA specimens were extracted and purified from peripheral leukocytes in dried-blood spotted on “Guthrie” filter cards, kindly provided by the local PRNSP, by using the Gentra^®^ Generation DNA Purification Kit (QIAGEN Inc., Valencia, CA, USA). For this purpose, the corresponding filter card punches were collected from an existing biobank repository of the PRNSP, following a controlled, stratified-by-regions, population-based sampling strategy in order to have specimens that were representative of the three distinct ethno-geographic regions of the island of Puerto Rico (West, Central and East). This sampling strategy also considered available regional birthrates to ensure the selection of at least one specimen from each of the hospitals in Puerto Rico with childbirth services, as documented on the 2004 National Birth Registry. DNA specimens were quantified using the NanoDrop™ 2000 Spectrophotometer (Thermo Scientific, Waltham, MA, USA). Working DNA stock samples were stored at −20 °C until their use. 

### 2.3. Genotyping Assay

Genotyping was performed by interrogating individual genomic DNA specimens for the following major allelic variants on the next gene loci, as described by the ClinVar website [19]: (1) *CYP2C19* (i.e., CYP2C19*2 (location at Chr10q23.3: 94781859 on assembly GRCh38, other names by HGVS: NM_000769.1(*CYP2C19*): c.681G > A (p.Pro227=), dbSNP:rs4244285) [20]; CYP2C19*3 (Chr10q23.3: 94780653, HGVS: NM_000769.1(*CYP2C19*): c.636G > A (p.Trp212Ter), dbSNP:rs4986893) [21]; CYP2C19*4 (Chr10q23.3: 94762706, HGVS:NM_000769.1(*CYP2C19*): c.1A > G (p.Met1Val), dbSNP:rs28399504) [22]; CYP2C19*17 (Chr10q23.3: 94761900, HGVS: NM_000769.1(*CYP2C19*): c.-806C > T, dbSNP:rs12248560) [23]; (2) *ABCB1* (Chr7q21.12: 87509329, HGVS: NM_000927.4(*ABCB1*): c.3435T > C (p.Ile1145=), dbSNP:rs1045642) [24]; (3) *P2RY12* (Chr3q24-q25.1: haplotype 2, defined by c.-15+742C > T intronic variant, rs2046934) [25] and (4) *PON1* (Chr7q21.3: 95308134, HGVS: NM_000446.6(*PON1*): c.575A > G (p.Gln192Arg), dbSNP:rs662, a.k.a. Q192R variant loci) [26]. *CYP2C19* alleles were also defined by specific SNPs according to the Pharmacogene Variation (PharmVar) Consortium [27]. 

Each test was run using the corresponding probe of commercial Taqman^®^-based SNP genotyping assays (Applied Biosystem, Foster City, CA, USA) and following the manufacturer’s instructions. Briefly, DNA amount ranged from 14 to 20 ng. The real-time PCR reactions took place in 96 or 48-well plates run in the Applied Biosystems Step-One plus Real-Time PCR system. Cycling parameters were 30 s at 60 °C, 10 min at 95 °C, 50 cycles of 15 s at 92 °C and 1 min at 60 °C, and a final step of 30 s at 60 °C, as previously described [18]. 

### 2.4. Statistical Analyses

The 95% confidence intervals (CI) for observed allele and genotype frequencies were calculated according to Newcombe and Altman’s method [28]. Allele and genotype proportions in our study cohort were compared versus those previously published in available databases for the following reference populations: (1) parental YRI (Yoruba population in Ibadan, Nigeria), *n* = 88: Yoruba in Ibadan with West-African ancestry, Nigeria; (2) parental CEU, *n* = 87: Utah residents with Northern and Western European ancestry from the CEPH (Centre d’Étude du Polymorphisme Humain) collection; (3) proxy of parental CHB, *n* = 97: Han Chinese in Beijing with Asian ancestry, China; and (4) comparable AMR (a composite of different Latino American populations), *n* = 181: a composite of different Latino American populations of Hispanic ethnicity such as Puerto Ricans in Puerto Rico (PUR), Colombian in Medellin, Colombia (CLM), Peruvian in Lima, Peru (PEL) and Mexican Ancestry in Los Angeles, CA, USA (MXL) from the 1000 Genome projects/Phase 3 [29,30,31,32]. A z-test for independent proportions was used to perform these comparisons. Departure from Hardy–Weinberg equilibrium (HWE) was tested under the null hypothesis of the predictable segregation ratio of specific matching genotypes (*p* > 0.05) by using the χ^2^ goodness-of-fit test or alternatively a binomial test if frequencies were lower than 5%. 

## 3. Results 

**Figure 1:**
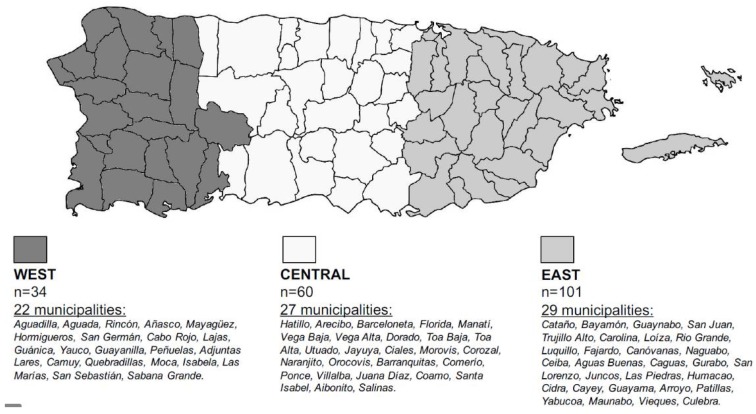
Ethno-geographic distribution of the total samples collected for the study. Sampling strategy was population-based and stratified-by-region, according to the 2004 National Registry of Births in the Commonwealth of Puerto Rico. *n* Indicates the number of samples collected per region. In parentheses, the number of municipalities from each region of the Island of Puerto Rico where at least one of the collected samples came from is indicated (i.e., 77 out of 78 municipalities are represented).

Figure 1 depicts the origin of all the genomic DNA specimens collected from the PRNSP biobank to perform the genetic screening in this study. Information was organized by ethno-geographic regions and includes specimens from 77 out of 78 municipalities in the Commonwealth of Puerto Rico, showing representability of the target population. Table 1 and Table 2 show the results of computed allele and genotype frequency distributions for all the genetic variants under consideration in this study. Although a total of 200 unrelated specimens were initially selected for the study, the prevalence of the two variants on *ABCB1* and *PON1*, was calculated in 197 samples; whereas for CYP2C19*3, CYP2C19*4, CYP2C19*2 and CYP2C19*17 the frequency was determined in 196 specimens. Finally, the *P2RY12* H2 prevalence was ascertained in 193 samples. These differences were due to non-calling, poor DNA quality, or low concentration.

### 3.1. ABCB1 Locus

Table 1 shows the results of allele and genotype frequencies of selected SNPs in Puerto Ricans. The observed MAF of the *ABCB1* c.3435T > C (p.Ile1145=, rs1045642) polymorphism was 41% (Table 2). Interestingly, this *ABCB1* c.3435C variant showed the highest frequency in the West (48%) and the lowest to the East (38%), suggesting a differential distribution or gradient across the Island (Supplemental Tables S1–S3). No significant deviation from Hardy–Weinberg equilibrium was found, according to the goodness-of-fit χ^2^-test (χ^2^ = 0.04, *p* > 0.05). The genotyping results for the *ABCB1* c.3435T > C variant in the study cohort showed that 69 out of 197 genomic DNA samples tested (35%) were found homozygous for the wild-type allele. Another 94 samples (47%) were single carriers for the allelic variant (heterozygous), whereas only 34 samples (17%) were found to be homozygous for this polymorphism. Distribution of double carriers for this variant across the Island also showed a distinctive pattern, with only 8% in the Central region and 29% to the West side. Single and double carriers combined (i.e., hetero- and homozygous for the variant) amounted to 68% and 72% of the samples in the West and Central regions, respectively, but represented only 59% of specimens collected from the East (Supplemental Tables S1–S3).

When compared to other reference populations (i.e., data taken from the 1000 Genomes project Phase 3) [30,31], we found that the calculated MAF in our target population was significantly different from those reported in two of the parental populations used as references (Table 2). Indeed, both YRI (African ancestry) and CEU (European ancestry) had MAF for this variant that significantly deviated from that observed in Puerto Ricans from our study cohort (i.e., 41% versus 12% and 59%, respectively; *p* < 0.01). On the other hand, neither MAFs of this variant in East Asians (CHB) and Latino Americans (AMR) (39% and 46%, respectively) nor those in the overall population (40%) were significantly different from the 41% in the study population. Notably, these findings were consistent with those observed across the three different ethno-geographic areas of the Island of Puerto Rico, after stratifying by regions (Supplemental Tables S4–S6).

### 3.2. PON1 Locus

The *PON1* polymorphism (c.575A > G, p.Gln192Arg, rs662, a.k.a. Q192R variant) was found to be highly frequent in our study samples, with a variant allelic frequency of 46% (Table 1). The genotyping results for *PON1* revealed that there are 59 homozygous for the common allele (WT), as well as 96 heterozygous and 42 homozygous for the variant allele among the 197 studied samples (i.e., 70% carriers). It is worth noting that the rs662 variant showed the highest frequency to the West (53%) and the lowest in the Central region (40%), suggesting again a different pattern of prevalence island-wide. Regional distribution of double carriers for this variant also reflected a particular pattern, with only 15% in Central municipalities and 35% to the West portion of the Island. Single and double carriers combined (dominance model) fluctuated between 71–74% of samples from the East and West regions and 65% of specimens from the center of the island (Supplemental Tables S1–S3).

The allele and genotype proportions of the *PON1* rs662 polymorphism in this study cohort were found to be in Hardy–Weinberg equilibrium (χ^2^ = 0.06, *p* > 0.05). When compared to other populations (Table 2), the frequency of the rs622A allele encoding for the 192R variant in our study cohort of PRNSP specimens (46%) was significantly higher than the corresponding frequency for this specific allele reported in Africans (YRI: 19%, *p* < 0.01), but lower than in Europeans (CEU, 69%, *p* = 0.0003). No significant differences were found for the allelic frequencies of *PON1* rs662 in East Asians (CHB: 40%, *p* = 0.33), Latinos from the Americas (AMR: 52%, *p* = 0.19) and the overall population (47%, *p* = 0.85) as compared to our study sample of PRNSP specimens. It is important to mention that both in Europeans (CEU) and Latinos (AMR) from the 1000 Genome project dataset, the ancestral rs622G allele is the minor allele at this locus, as opposed to what we found in Puerto Ricans from the PRNSP (ancestral allele frequency of 54%). Although slightly higher, the observed prevalence for this ancestral allele in PRNSP specimens is quite similar to those previously reported in 126 Latin America inhabitants (i.e., Mexicans and Colombians in the 1000 Genomes project, Phase I selection), 58 Mexican descendants who reside in Los Angeles, California (HapMap-MEX; HapMap project dataset, release#28, Phase II + III) and the Puerto Ricans in the 1000 Genomes project, Phase III selection, where the *PON1* rs662G allele frequencies were estimated to be 47%, 50% and 48%, respectively [31]. After stratifying by geographic regions, significant differences of allelic frequencies remain for the comparison between our PRNSP cohort and both YRI and CEU populations, but not versus CHB, AMR and the overall population (Supplemental Tables S4–S6). 

### 3.3. P2RY12 Locus

The MAF of the allelic variant defining the *P2RY12* H2 haplotype (rs2046934C > T) was found to be 12.4% in the study cohort of specimens from PRNSP. No significant deviation from Hardy–Weinberg equilibrium was found, according to the goodness-of-fit χ^2^-test (χ^2^ = 2.73, *p* > 0.05). The genotyping results for this locus showed that 152 out of the 193 tested gDNA samples (78%) were homozygous for the wild-type allele, whereas 41 samples (21%) were single carrier (heterozygous) but no homozygote of the allelic variant were found. Comparison of the results with other populations found no significant differences between our findings in the study samples and those earlier reported in reference populations, even after stratifying by regions (Supplemental Tables S4–S6). Furthermore, no substantial differences of MAFs among the three geographic regions were found (range: 10–11%), nor were the number of heterozygous across the three zones found to be significantly divergent one other (i.e., 20–22%). 

### 3.4. CYP2C19 Locus

Four major SNPs occurring at the *CYP2C19* locus were included in the study (i.e., CYP2C19*2, *3, *4 and the gain-of-function (GOF) *17 allele). The computed MAFs of the CYP2C19*3 and *4 variants in the study population were 0 and 0.003, respectively. In fact, only one specimen from the East region tested positive for the CYP2C19*4 allele (heterozygous). Concerning the CYP2C19*3, this loss-of-function (LOF) polymorphism has a reported a prevalence of only 1% in the general population (Table 2) but seems to be only present in individuals of East Asian ancestry (MAF = 0.046 in CHB versus 0 in the other reference populations, *p* < 0.01). Regarding the CYP2C19*4 allele, no significant differences were detected after comparing the MAFs of this variant in Puerto Ricans from the PRNSP versus those reported in four reference populations from the 1000 Genome project, before and after sorting samples out by regions (Table 2 and Supplemental Tables S4–S6). 

As expected, both the LOF CYP2C19*2 and the GOF CYP2C19*17 alleles had higher MAFs in the study population, with frequencies of 13.5% and 14.1%, respectively. The genotyping call results for the CYP2C19*2 allele showed that 145 out of 196 samples (74%) were tested as homozygous for the WT allele, another 49 samples (25%) were single carriers of the *2 allele (heterozygous), whereas only two specimens (1%) were homozygous of this dysfunctional variant. For the GOF CYP2C19*17 allele in the promoter region of the gene, it was found that 144 out of 195 samples (74%) were tested as homozygous for the WT allele; another 47 samples (24%) were single carriers of the allelic variant (heterozygous) and the remaining four specimens (2%) were homozygous of the GOF allele (Table 1). No significant deviation from Hardy–Weinberg equilibrium was found in the reported proportions for any of these polymorphisms (*p* > 0.05). When stratified by geographic regions, the allelic frequency of CYP2C19*2 was found to be the lowest in the Central region (11.7%) as compared to 13.6% in the West and up to 15% to the East side (highest prevalence). The number of carriers (single and double carriers combined) for the *2 allele was 51 overall (26%), which ranged from 23 to 27% across the island (Table 1 and Supplemental Tables S1–S3). On the other hand, the CYP2C19*17 MAFs by regions fluctuated from 12.5% (West and Central) to the highest 16% in the East portion of the Island. The number of carriers (combinatorial homozygous + heterozygous) of this variant was approximately 22% in the West and Central but increase to about 30% to the East (Supplemental Tables S1–S3).

Both variants showed frequencies in Puerto Ricans from the PRNSP that were found to be significant differences from that previously reported in individuals with East Asian ancestry (Table 2). These statistical differences remained significant after stratifying by regions (Supplemental Tables S4–S6). In addition, the MAF of CYP2C19*17 in Puerto Ricans was also found to be different to that in YRI (African ancestry, Table 2) when performing the comparison for the total cohort of 195 specimens (i.e., 0.141 versus 0.256, *p* = 0.019), but this observed difference did not sustain after stratifying the cohort by regions (Supplemental Tables S4–S6).

## 4. Discussion

This cross-sectional study was performed to assess the frequency distribution of seven relevant pharmacogenetics markers in four candidate pharmacogenes associated with clopidogrel effectiveness. To the best of our knowledge, this is the first ever report of clopidogrel-associated alleles and genotypes frequency distributions in the Puerto Rican population that takes into consideration variability across the three major ethno-geographic regions of the Commonwealth of Puerto Rico (i.e., East, Central and West regions). This is of remarkable interest in the pharmacogenetic characterization of clopidogrel given the particular stratification of the population across the Island [33]. This attribute, along with the high genetic heterogeneity and admixture pattern in Puerto Ricans, has the potential to enhance the expected diversity within Puerto Rico. The selection of a population-based rather than a household strategy of sampling reduced the effect of cryptic relatedness and subsequent increment of homozygosity in future data analyses. This design enabled to identify important differences in frequency distribution between our study and the Puerto Rican sample included in the 1000 Genome project, the latter mostly used a household sampling strategy that may have enhanced the recruitment of relatives (a bias in the experimental design).

This study also expands our preliminary findings from previous pilot, exploratory protocols of clopidogrel-associated alleles and genotypes prevalence in Puerto Ricans from the PRNSP [18,34]. In these preliminary studies, 99 and 122 specimens from the PRNSP were genotyped by Taqman^®^ Genotyping Assays [18] and a combination of RFLP-PCR method and physiogenomic (PG)-array through Illumina BeadArrayTM technology, respectively [34]. In the study with the larger sample size (*n* = 122), we found a CYP2C19*2 MAF of 14%, with 24.6% of single-carriers and 1.64% of homozygous for the variants [34]. Data from the present study (i.e., CYP2C19*2 MAF = 13.5%, with 25% of carriers and 1% homozygous, in Table 1) replicates this prior result, including the absence of carriers for the LOF CYP2C19*3 variant (MAF = 0). In the other prior study (*n* = 99), the analysis revealed a slightly lower but not statistically different MAF of 9% [18]. However, because these two initial studies included fewer than 150 specimens, they fell short of an adequate sample size and, therefore, the statistical power to detect the actual prevalence in the Commonwealth of Puerto Rico. Despite these limitations, these preliminary data were useful to calculate a sample size of at least 180 specimens to perform a proper characterization of prevalence for these relevant variants in Puerto Ricans. Accordingly, the present study is powered enough to validate our prior findings and serves as a confirmatory survey of the earlier evaluations. 

In two other studies, Duconge et al. (2008; 2012) also reported no carriers of the CYP2C19*3 variant in Puerto Ricans [34,35]. Like CYP2C19*3, the CYP2C19*2 variant is more common among individuals of East and South/Central Asian ancestry, where a larger number of carriers has been reported (CYP2C19*2 MAF = 29% and 35%, respectively, and up to 15% of homozygous) [17]. Accordingly, the CYP2C19*2 MAF in Puerto Ricans from the PRNSP was found to be significant difference from the corresponding frequency in CHB (*p* = 0.0002, Table 2). Based on previous reports, genetically-inferred poor metabolizers (i.e., CYP2C19*2/*2 homozygous) in both European and African populations is known to range from 2 to 5%, with MAFs of 15% [17]. Although the percentage of homozygous (1–1.6%) and the MAF in Puerto Ricans were indeed lower than those in European and Africans, these differences did not meet the threshold of statistical significance. In addition, our results confirmed that CYP2C19*2 is more prevalent than CYP2C19*3 among Puerto Ricans. This is consistent with data from the 1000 Genome project and other available databases where the rare CYP2C19*3 allele is not usually found outside of the East Asian population [31].

As expected, the rare CYP2C19*4 variant was found at a very low MAF of 0.3% in our study. Only one specimen (heterozygous) was identified as carrier of this allele. This frequency was slightly lower than that reported for Latinos in the 1000 Genome Project database, which includes 134 Puerto Ricans, though no significant difference was found (MAF = 0.6% versus 0.3%, *p* = 0.61). No other reports of CYP2C19*4 prevalence in Puerto Ricans are available.

Regarding the GOF CYP2C19*17 allele, MAF differences were found between Puerto Ricans and both CHB (*p* = 0.013) and YRI (*p* = 0.019) populations, with lower allele prevalence in Puerto Ricans (26% versus 14%). This unique frequency distribution pattern of allelic variants at the same *CYP2C19* locus on chromosome 10 can be partially explained by the high degree of admixture within Puerto Ricans. The Puerto Rico population has a complex genomic structure, with a rich repertoire of genetic combinations and a trichotomous pattern of relatively recent mixture of Caucasians (mostly Iberians), West-Africans, and Amerindians (Tainos Arawaks). This high content of admixture and the unique allelic frequency distributions arose from a history of about 500 years of migrations, founder effects, genetic drifts, and ancestral contributions of at least three major parental populations at varying degree across the Island [36]. Consequently, it has shaped a mosaic of highly heterogeneous haplotype blocks and genotypes that merits special evaluation for their potential impact on drug metabolism and response (effectiveness and safety). 

The *PON1* Q192R polymorphism has been previously linked to clopidogrel response variability, though with mixed conclusions [37,38,39]. Data from our lab has previously described an overall allele frequency of 45% for this variant in Puerto Ricans [18], and the present study confirms this report (MAF = 46%, Table 1). The clinical significance of this polymorphism resides on the potential association of the gene product with clopidogrel biotransformation (i.e., an alternate pathway mediated by this paraoxonase/arylesterase enzyme) as well as the suggested role of the *PON1* 192R variant as a risk factor for ischemic stroke and heart diseases [40]. Bouman et al., argued that homozygotes for the mutation variant 192R have lower *PON1* activity which is postulated to be associated with a reduced platelet inhibition and higher risk for stent thrombosis in percutaneous coronary intervention (PCI) patients [37]. This conclusion has been challenged by several studies that have failed in replicating these results and demonstrated no clear association [39]. Unfortunately, no study to date has evaluated the genetic contribution of *PON1* polymorphisms on clopidogrel resistance in Puerto Ricans.

The *ABCB1* c.3435T > C (p.Ile1145=), a.k.a. rs1045642, variant encodes a drug-efflux transporter MDR1/P-glycoprotein that is directly involved in the intestinal absorption of clopidogrel [41]. This relevant protein is mainly present in the duodenum and the expression of its homozygous variant has been linked to a decreased oral clopidogrel bioavailability, platelet inhibition, and cardiovascular complications [42]. The available data from different ethnic populations worldwide have demonstrated a varying percentage of allele proportions [43]. This study found a MAF of 0.41 for this variant that differed only from the corresponding MAFs reported in Africans and Europeans from the 1000 Genome Project database. Only one study evaluated the implications of *ABCB1* c.3435T > C polymorphism in a Hispanic population [44]. Interestingly, the authors also found an *ABCB1* c.3435T > C MAF of 0.41 and reported a direct association of this SNP with decreased clopidogrel responsiveness. No early reports describing the c.3435T > C polymorphism in the admixed Puerto Rican population are available. Hence, this study is the first one describing the frequency of *ABCB1* c.3435T > C polymorphism in our target population. 

The *P2RY12* gene encodes the clinically relevant P2Y12 receptor located on the surface of platelets that is responsible for platelet aggregation [45]. Polymorphisms occurring on this locus have been studied for their possible association with HTPR in patients on clopidogrel. However, results from previous studies are inconsistent and controversial [46,47,48]. The MAF of the allele defining the *P2RY12* H2 haplotype was 0.159 in the general population, which was not significantly different from the MAF of 0.124 in our cohort of Puerto Ricans (*p* = 0.26, Table 2). Data from a large observational study in a German population revealed an H2 allele frequency of 0.154 with 2.5% of the studied patients being homozygote carriers of the H2 haplotype [49]. This last group exhibited significantly enhanced platelet reactivity which is translated into clopidogrel resistance. 

## 5. Study Limitations

While this study used a population-based, stratified-by-region sampling strategy to be representative of the Puerto Rican population, the number of individuals within each region was limited and should be considered as exploratory. Furthermore, relatively recent migration patterns within the Commonwealth of Puerto Rico may also have a confounding effect on the region-specific analyses. Nonetheless, we believe the selection of a population-based strategy including samples from each of the hospitals in Puerto Rico with childbirth services, rather than a household strategy of sampling, provides strength in the overall representative validity of the study. 

## 6. Conclusions

In summary, we report the prevalence of seven major allelic variants and resulting genotypes in four relevant pharmacogenes for clopidogrel responsiveness (*ABCB1*, *PON1*, *CYP2C19* and *P2RY12*) in Puerto Rico. Some of these variants showed a unique geographic distribution. These prevalence data provide a necessary groundwork for future clinical studies of clopidogrel pharmacogenetics in Caribbean Hispanics.

## Figures and Tables

**Table 1:** Overall genotype and allelic frequencies of seven relevant polymorphisms in four pharmacogenes of interest (i.e., potentially to be associated with clopidogrel response) in the Puerto Rican population. Data correspond to genomic DNA specimens from the Puerto Rico newborn screening program (PRNSP) that were collected from different ethno-geographic regions across the Island of Puerto Rico. Totals are less than 200 due to poor DNA quality or non-calling. * tagSNP for calling (defining) the *P2RY12* haplotype 2.

Status/Marker	*ABCB1* (C > T)		*PON1* (G > A)		CYP2C19*3 (G > A)		CYP2C19*4 (A > G)		CYP2C19*2 (G > A)		CYP2C19*17 (C > T)		*P2RY12* H2 (C > T) *	
	No.	Freq.	No.	Freq.	No.	Freq.	No.	Freq.	No.	Freq.	No.	Freq.	No.	Freq.
**Genotypes**														
Homozygous wild-type (WT)	69	0.350	59	0.299	196	1.000	195	0.995	145	0.739	144	0.738	152	0.786
Heterozygous	94	0.477	96	0.487	0	0	1	0.005	49	0.250	47	0.241	41	0.212
Homozygous Variant	34	0.173	42	0.213	0	0	0	0	2	0.010	4	0.021	0	0
Totals (genotypes counts/freq.)	197	1.000	197	1.000	196	1.000	196	1.000	196	1.000	195	1.000	193	1.000
**Alleles**														
Minor allele (variant)	162	0.411	180	0.457	0	0	1	0.003	53	0.135	55	0.141	48	0.124
Totals (allele counts/freq.)	394	1.000	394	1.000	392	1.000	392	1.000	392	1.000	390	1.000	386	1.000

Note: Freq. means frequency of alleles or genotypes.

**Table 2:** Comparisons of minor allele frequencies (MAFs) between the study cohort and all the parental/reference populations of the 1000 Genome Project/Phase 3. The corresponding *p*-values are depicted in the column named “Sign.”, where an asterisk is added to indicate statistical significance (*p* < 0.05). MAF_PR_ stands for observed minor allele frequency in Puerto Ricans from the study cohort.

Populations/Marker	*ABCB1* *N* = 197; (MAF_PR_: 0.411)		*PON1* *N* = 197; (MAF_PR_: 0.457)		CYP2C19*3 *N* = 196; (MAF_PR_: 0.000)		CYP2C19*4 *N* = 196; (MAF_PR_: 0.003)		CYP2C19*2 *N* = 196; (MAF_PR_: 0.135)		CYP2C19*17 *N* = 195; (MAF_PR_: 0.141)		*P2RY12* H2 ** N = 193; (MAF_PR_: 0.124)	
	MAF	Sign.	MAF	Sign.	MAF	Sign.	MAF	Sign.	MAF	Sign.	MAF	Sign.	MAF	Sign.
1000 Genome Project Reference Populations														
YRI (*n* = 88)	0.119	<0.01 *	0.193	<0.01 *	0.000	-	0.000	0.630	0.165	0.510	0.256	0.019 *	0.170	0.300
CEU (*n* = 87)	0.414 ^#^	<0.01 *	0.310 ^¶^	<0.01 *	0.000	-	0.000	0.630	0.138	0.950	0.224	0.083	0.195	0.120
CHB (*n* = 97)	0.397	0.820	0.397	0.330	0.046	<0.01 *	0.005	0.740	0.320	<0.01 *	0.260	0.013 *	0.211	0.053
AMR (*n* = 181)	0.461	0.327	0.475 ^¶^	0.190	0.000	-	0.006	0.610	0.133	0.950	0.116	0.471	0.108	0.620
Overall Population (*n* = 453)	0.405	0.880	0.465	0.850	0.010	0.160	0.003	0.930	0.180	0.160	0.145	0.896	0.159	0.260

^#^ C is the minor allele at this locus in Europeans; ^¶^ G is the minor allele at this locus in Europeans and Latinos; ** This is the tagSNP for calling (defining) the *P2RY12* haplotype 2. * means statistically significant difference. YRI: Yoruba in Ibadan, Nigeria; CEU: Utah residents with Northern and Western European ancestry from the CEPH collection; CHB: Han Chinese in Beijing, China; AMR: a composite of different Latino American populations of Hispanic ethnicity.

## Supplementary Materials

The following are available online at http://www.mdpi.com/1660-4601/15/6/1115/s1. Table S1: Genotype and allelic frequencies of seven relevant polymorphisms occurring on four pharmacogenes of interest (i.e., potentially to be associated with clopidogrel response) in the Puerto Rican population. Data correspond to genomic DNA specimens from the PRNSP that were collected from West region of the Island of Puerto Rico. Totals are less than expected due either to poor DNA quality or non-calling. * tagSNP for calling (defining) the *P2RY12* haplotype 2. Table S2: Genotype and allelic frequencies of seven relevant polymorphisms occurring on four pharmacogenes of interest (i.e., potentially to be associated with clopidogrel response) in the Puerto Rican population. Data correspond to genomic DNA specimens from the PRNSP that were collected from Central region of the Island of Puerto Rico. Totals are less than expected due either to poor DNA quality or non-calling. * tagSNP for calling (defining) the *P2RY12* haplotype 2. Table S3: Genotype and allelic frequencies of seven relevant polymorphisms occurring on four pharmacogenes of interest (i.e., potentially to be associated with clopidogrel response) in the Puerto Rican population. Data correspond to genomic DNA specimens from the PRNSP that were collected from East region of the Island of Puerto Rico. Totals are less than expected due either to poor DNA quality or non-calling. * tagSNP for calling (defining) the *P2RY12* haplotype 2. Table S4: Comparisons of minor allele frequencies (MAFs) between the study cohort (West Region) and all the parental/ reference populations of the 1000 Genome Project/Phase 3 at seven locus of interest selected for this study. The corresponding *p*-values are given in the column named “Sign.”, where an asterisk is added to indicate statistical significance (*p* < 0.05). MAFPR stands for observed minor allele frequency in Puerto Ricans from the study cohort. Table S5: Comparisons of minor allele frequencies (MAFs) between the study cohort (Central Region) and all the parental/ reference populations of the 1000 Genome Project/Phase 3 at seven locus of interest selected for this study. The corresponding *p*-values are given in the column named “Sign.”, where an asterisk is added to indicate statistical significance (*p* < 0.05). MAFPR stands for observed minor allele frequency in Puerto Ricans from the study cohort. Table S6: Comparisons of minor allele frequencies (MAFs) between the study cohort (East Region) and all the parental/reference populations of the 1000 Genome Project/Phase 3 at seven locus of interest selected for this study. The corresponding *p*-values are given in the column named “Sign.”, where an asterisk is added to indicate statistical significance (*p* < 0.05). MAFPR stands for observed minor allele frequency in Puerto Ricans from the study cohort.
