# Supplementary material for: Clinical Relevant Polymorphisms Affecting Clopidogrel Pharmacokinetics and Pharmacodynamics: Insights from the Puerto Rico Newborn Screening Program
Source: Int J Environ Res Public Health. 2018 May 30;15(6):1115. doi: 10.3390/ijerph15061115 (PMC6025039; doi:10.3390/ijerph15061115)
Supplement: Supplementary file 1 [file ijerph-15-01115-s001.pdf]

Table S1. Genotype and allelic frequencies of seven relevant polymorphisms occurring on four pharmacogenes of interest (i.e., potentially to be associated with clopidogrel response) in the Puerto Rican population. Data correspond to genomic DNA specimens from the PRNSP that were collected from *West* region of the Island of Puerto Rico. Totals are less than expected due either to poor DNA quality or non-calling. * tagSNP for calling (defining) the *P2RY12* haplotype 2.

**Status/Markers**	***ABCB1*** (C > T)		***PON1*** (G > A)		***CYP2C19**3** (G > A)		***CYP2C19**4** (A > G)		***CYP2C19**2** (G > A)		***CYP2C19**17** (C > T)		***P2RY12* H2** (C > T) *	
	**No.**	**Freq.**	**No.**	**Freq.**	**No.**	**Freq.**	**No.**	**Freq.**	**No.**	**Freq.**	**No.**	**Freq.**	**No.**	**Freq.**
**Genotypes**														
Homozygous wild-type (WT)	11	0.324	10	0.294	33	1.000	33	1.000	24	0.727	25	0.781	24	0.800
Heterozygous	13	0.382	12	0.353	0	0	0	0	9	0.273	6	0.188	6	0.200
Homozygous Variant	10	0.294	12	0.353	0	0	0	0	0	0	1	0.031	0	0
Totals (genotypes counts/freq.)	34	1.000	34	1.000	33	1.000	33	1.000	33	1.000	32	1.000	30	1.000
**Alleles**														
Minor allele (variant)	33	0.485	36	0.529	0	0	0	0	9	0.136	8	0.125	6	0.100
Totals (allele counts/freq.)	68	1.000	68	1.000	66	1.000	66	1.000	66	1.000	64	1.000	60	1.000

Table S2. Genotype and allelic frequencies of seven relevant polymorphisms occurring on four pharmacogenes of interest (i.e., potentially to be associated with clopidogrel response) in the Puerto Rican population. Data correspond to genomic DNA specimens from the PRNSP that were collected from *Central* region of the Island of Puerto Rico. Totals are less than expected due either to poor DNA quality or non-calling. * tagSNP for calling (defining) the *P2RY12* haplotype 2.

**Status/Markers**	***ABCB1*** (C > T)		***PON1*** (G > A)		***CYP2C19**3** (G > A)		***CYP2C19**4** (A > G)		***CYP2C19**2** (G > A)		***CYP2C19**17** (C > T)		***P2RY12* H2** (C > T) *	
	**No.**	**Freq.**	**No.**	**Freq.**	**No.**	**Freq.**	**No.**	**Freq.**	**No.**	**Freq.**	**No.**	**Freq.**	**No.**	**Freq.**
**Genotypes**														
Homozygous wild-type (WT)	17	0.283	21	0.350	60	1.000	60	1.000	46	0.767	46	0.767	47	0.783
Heterozygous	38	0.633	30	0.500	0	0	0	0	14	0.233	13	0.217	13	0.217
Homozygous Variant	5	0.083	9	0.150	0	0	0	0	0	0	1	0.017	0	0
Totals (genotypes counts/freq.)	60	1.000	60	1.000	60	1.000	60	1.000	60	1.000	60	1.000	60	1.000
**Alleles**														
Minor allele (variant)	48	0.400	48	0.400	0	0	0	0	14	0.117	15	0.125	13	0.108

**Totals (allele counts/freq.)**	120	1.000	120	1.000	120	1.000	120	1.000	120	1.000	120	1.000	120	1.000

Table S3. Genotype and allelic frequencies of seven relevant polymorphisms occurring on four pharmacogenes of interest (i.e., potentially to be associated with clopidogrel response) in the Puerto Rican population. Data correspond to genomic DNA specimens from the PRNSP that were collected from East region of the Island of Puerto Rico. Totals are less than expected due either to poor DNA quality or non-calling. * tagSNP for calling (defining) the P2RY12 haplotype 2.

**Status/Markers**	***ABCB1*** (C > T)		***PON1*** (G > A)		***CYP2C19*3*** (G > A)		***CYP2C19*4*** (A > G)		***CYP2C19*2*** (G > A)		***CYP2C19*17*** (C > T)		***P2RY12* H2** (C > T) *	
	**No.**	**Freq.**	**No.**	**Freq.**	**No.**	**Freq.**	**No.**	**Freq.**	**No.**	**Freq.**	**No.**	**Freq.**	**No.**	**Freq.**
**Genotypes**														
Homozygous wild-type (WT)	41	0.406	26	0.267	101	1.000	100	0.990	73	0.723	71	0.703	78	0.780
Heterozygous	43	0.426	54	0.535	0	0	1	0.009	26	0.257	28	0.277	22	0.220
Homozygous Variant	17	0.168	21	0.208	0	0	0	0	2	0.020	2	0.020	0	0
Totals (genotypes counts/freq.)	101	1.000	101	1.000	101	1.000	101	1.000	101	1.000	101	1.000	100	1.000
**Alleles**														
Minor allele (variant)	77	0.382	96	0.475	0	0	1	0.005	30	0.149	32	0.158	22	0.110
Totals (allele counts/freq.)	202	1.000	202	1.000	202	1.000	202	1.000	202	1.000	202	1.000	200	1.000

Table S4. Comparisons of minor allele frequencies (MAFs) between the study cohort (West Region) and all the parental/ reference populations of the 1000 Genome Project/Phase 3 at seven locus of interest selected for this study. The corresponding *p*-values are given in the column named "Sign.", where an asterisk is added to indicate statistical significance ($p < 0.05$). MAF$_{PR}$ stands for observed minor allele frequency in Puerto Ricans from the study cohort.

**Population/Markers**	***ABCB1*** $N = 34$; (MAF$_{PR}$: 0.4853)		***PON1*** $N = 34$; (MAF$_{PR}$: 0.5294)		***CYP2C19*3*** $N = 33$; (MAF$_{PR}$: 0)		***CYP2C19*4*** $N = 33$; (MAF$_{PR}$: 0)		***CYP2C19*2*** $N = 33$; (MAF$_{PR}$: 0.136)		***CYP2C19*17*** $N = 32$; (MAF$_{PR}$: 0.125)		***P2RY12* H2**** $N = 30$; (MAF$_{PR}$: 0.100)	
	MAF (A)	Sign.	MAF (T)	Sign.	MAF (A)	Sign.	MAF (G)	Sign.	MAF (A)	Sign.	MAF (T)	Sign.	MAF (G)	Sign.
**1000 Genome Project Reference Populations**														
YRI (n=88)	0.119	<0.01 *	0.193	<0.01 *	0.000	-	0.000	-	0.165	0.704	0.256	0.126	0.170	0.357
CEU (n=87)	0.414 #	0.317	0.310 ¶	0.097	0.000	-	0.000	-	0.138	0.984	0.224	0.230	0.195	0.234
CHB (n=97)	0.397	0.368	0.397	0.180	0.046	0.211	0.005	0.682	0.320	0.041 *	0.260	0.026 *	0.211	0.171
AMR (n=181)	0.461	0.794	0.475 ¶	0.960	0.000	-	0.006	0.653	0.133	0.960	0.116	0.881	0.108	0.896

Overall Population (n=453)	0.405	0.357	0.465	0.465	0.010	0.562	0.003	0.749	0.180	0.529	0.145	0.756	0.159	0.389

Notes: # G is the minor allele at this locus in Europeans; ¶ C is the minor allele at this locus in Europeans and Latinos; ** This is the tagSNP for calling (defining) the P2RY12 haplotype 2. * means statistically significant difference.

Table S5. Comparisons of minor allele frequencies (MAFs) between the study cohort (Central Region) and all the parental/ reference populations of the 1000 Genome Project/Phase 3 at seven locus of interest selected for this study. The corresponding *p*-values are given in the column named "Sign.", where an asterisk is added to indicate statistical significance ($p < 0.05$). MAF$_{PR}$ stands for observed minor allele frequency in Puerto Ricans from the study cohort.

Population/Markers	**ABCB1** *N* = 60; (MAF$_{PR}$: 0.400)		**PON1** *N* = 60; (MAF$_{PR}$: 0.400)		**CYP2C19*3** *N* = 60; (MAF$_{PR}$: 0)		**CYP2C19*4** *N* = 60; (MAF$_{PR}$: 0)		**CYP2C19*2** *N* = 60; (MAF$_{PR}$: 0.117)		**CYP2C19*17** *N* = 60; (MAF$_{PR}$: 0.125)		**P2RY12 H2**** *N* = 60; (MAF$_{PR}$: 0.108)	
	MAF (A)	Sign.	MAF (T)	Sign.	MAF (A)	Sign.	MAF (G)	Sign.	MAF (A)	Sign.	MAF (T)	Sign.	MAF (G)	Sign.
**1000 Genome Project Reference Populations**														
YRI (*n* = 88)	0.119	<0.01 *	0.193	<0.01 *	0.000	-	0.000	-	0.165	0.412	0.256	0.051	0.170	0.293
CEU (*n* = 87)	0.414 #	0.026 *	0.310 ¶	<0.01 *	0.000	-	0.000	-	0.138	0.704	0.224	0.128	0.195	0.158
CHB (*n* = 97)	0.397	0.968	0.397	0.968	0.046	0.091	0.005	0.582	0.320	<0.01 *	0.260	0.013 *	0.211	0.097
AMR (*n* = 181)	0.461	0.412	0.475 ¶	0.093	0.000	-	0.006	0.548	0.133	0.741	0.116	0.849	0.108	0.992
Overall Population (*n* = 453)	0.405	0.944	0.465	0.342	0.010	0.435	0.003	0.675	0.180	0.223	0.145	0.674	0.159	0.308

Notes: # G is the minor allele at this locus in Europeans; ¶ C is the minor allele at this locus in Europeans and Latinos; ** This is the tagSNP for calling (defining) the P2RY12 haplotype 2. * means statistically significant difference.

Table S6. Comparisons of minor allele frequencies (MAFs) between the study cohort (East Region) and all the parental/ reference populations of the 1000 Genome Project/Phase 3 at seven locus of interest selected for this study. The corresponding *p*-values are given in the column named "Sign.", where an asterisk is added to indicate statistical significance ($p < 0.05$). MAF$_{PR}$ stands for observed minor allele frequency in Puerto Ricans from the study cohort.

Population/Markers	**ABCB1** *N* = 101; (MAF$_{PR}$: 0.381)		**PON1** *N* = 101; (MAF$_{PR}$: 0.475)		**CYP2C19*3** *N* = 101; (MAF$_{PR}$: 0)		**CYP2C19*4** *N* = 101; (MAF$_{PR}$: 0.005)		**CYP2C19*2** *N* = 101; (MAF$_{PR}$: 0.148)		**CYP2C19*17** *N* = 101; (MAF$_{PR}$: 0.158)		**P2RY12 H2**** *N* = 100; (MAF$_{PR}$: 0.110)	
	MAF (A)	Sign.	MAF (T)	Sign.	MAF (A)	Sign.	MAF (G)	Sign.	MAF (A)	Sign.	MAF (T)	Sign.	MAF (G)	Sign.
**1000 Genome Project Reference Populations**														

YRI (*n* = 88)	0.119	<0.01 *	0.193	<0.01 *	0.000	-	0.000	0.509	0.165	0.756	0.256	0.097	0.170	0.234
CEU (*n* = 87)	0.414 #	<0.01 *	0.310 ¶	<0.01 *	0.000	-	0.000	0.509	0.138	0.841	0.224	0.250	0.195	0.103
CHB (*n* = 97)	0.397	0.818	0.397	0.267	0.046	0.293	0.005	0.999	0.320	<0.01 *	0.260	<0.01 *	0.211	0.053
AMR (*n* = 181)	0.461	0.193	0.475 ¶	0.423	0.000	-	0.006	0.912	0.133	0.718	0.116	0.312	0.108	0.960
Overall Population (*n* = 453)	0.405	0.659	0.465	0.849	0.010	0.312	0.003	0.756	0.180	0.453	0.145	0.728	0.159	0.215

Notes: # G is the minor allele at this locus in Europeans; ¶ C is the minor allele at this locus in Europeans and Latinos; ** This is the tagSNP for calling (defining) the P2RY12 haplotype 2. * means statistically significant difference.
